# Catastrophic Health Expenditures for In-State and Out-of-State Abortion Care

**DOI:** 10.1001/jamanetworkopen.2024.44146

**Published:** 2024-11-08

**Authors:** Ortal Wasser, Lauren J. Ralph, Shelly Kaller, M. Antonia Biggs

**Affiliations:** 1 Silver School of Social Work, New York University, New York; 2 Advancing New Standards in Reproductive Health, University of California San Francisco, Oakland

## Abstract

**Importance:**

Most US individuals who access abortion care pay out of pocket due to insurance coverage restrictions on abortion. More research is needed on the financial and psychological burdens of abortion seeking, particularly for those traveling across state lines for care.

**Objectives:**

To estimate the proportion of patients seeking abortion who incur abortion-related catastrophic health expenditures (CHEs), assess whether CHE differs between those seeking care in state vs out of state, and examine the association of CHE with mental health symptoms.

**Design, Setting, and Participants:**

In this cross-sectional study conducted before the *Dobbs v Jackson Women’s Health Organization* decision, surveys were administered between January and June 2019 among individuals aged 15 to 45 years seeking abortion in 4 clinics located in abortion-supportive states (California, Illinois, and New Mexico). Participants completed self-administered questionnaires while awaiting their abortion appointment. Analyses were conducted from November 2023 to April 2024.

**Exposure:**

Travel for abortion care, categorized as either out of state or in state based on participants’ state of residence and the clinic location.

**Main Outcomes and Measures:**

Self-reported abortion care costs and additional non–health care costs (eg, transportation, accommodation, and missed work), which were considered catastrophic if they were 40% or greater of participants’ ability to pay (defined as monthly income remaining after meeting subsistence needs). Multivariable regression analyses were conducted to examine associations between CHE, out-of-state travel for abortion care, and mental health symptoms including stress, anxiety, and depression.

**Results:**

Among the 675 participants included in the analytic sample, mean (SD) age was 27.33 (6.27) years; most were in their 20s (374 [55%]), and all but 196 (29%) sought abortion before or at 12 weeks’ gestation. A total of 285 participants (42%) were estimated to incur abortion-related CHEs, which was associated with anxiety (APR, 1.13; 95% CI, 1.07-1.19) and depression (APR, 1.25; 95% CI, 1.12-1.39). Of people traveling from out of state (212 [31%]), more were likely to incur CHEs (138 [65%]) compared with those seeking care in state (147 of 463 [32%]) (APR, 2.24; 95% CI, 1.67-3.00).

**Conclusions and Relevance:**

In this cross-sectional study of US patients seeking abortion, many individuals and their households were estimated to incur CHEs, particularly those traveling from out of state. The financial and psychological burdens of abortion seeking have likely worsened after the *Dobbs* decision, as more people need to cross state lines to reach abortion care. The findings suggest expansion of insurance coverage to ensure equitable access to abortion care, irrespective of people’s state of residence, is needed.

## Key Points

**Question:**

What proportion of individuals accessing abortion care incur abortion-related catastrophic health expenditures (CHEs), and does CHE differ between those seeking care in state vs out of state?

**Findings:**

In this cross-sectional study of 675 US patients seeking abortion before the *Dobbs v Jackson Women’s Health Organization* decision, 42% of participants were estimated to incur abortion-related CHEs. A significantly higher proportion of people traveling from out of state were likely to incur abortion-related CHEs (65%) compared with those seeking care in state (32%).

**Meaning:**

The study’s finding of a high rate of abortion-related CHE, particularly among people traveling from out of state, suggests a need for expanded insurance coverage for abortion care, regardless of state of residence.

## Teaser

This cross-sectional study estimates the prevalence of catastrophic health expenditures incurred by US individuals accessing abortion care and whether abortion costs differ among those seeking care within or outside their state of residence.

## Introduction

Most patients who seek abortion care in the US pay out of pocket for care, irrespective of their health insurance.^1,2,3,4^ The Hyde Amendment bans the use of federal Medicaid funds for abortions, and state-level policies restrict private insurance coverage for these services.^3,5^ Abortion is also an inherently unexpected expense for time-sensitive medical care, requiring individuals to gather funds quickly. Prolonged care-seeking inevitably leads to further expenses, since the cost of abortion rises with advancing gestation: the median cost ranges from approximately $560 for abortion in the first trimester to $895 in the second trimester.^4,6^ However, these prices do not account for the total financial burden faced by pregnant people when they seek care. Restrictive abortion policies around the US force many people to cross state lines and travel considerable distances to reach a clinic.^7,8,9,10^ As a result, in addition to the cost of the abortion itself, those who travel for care often incur substantial ancillary non–health care expenses such as transportation, accommodation, child care, and lost wages.^7,11,12^ These health care–seeking expenditures could be an insurmountable hardship for many pregnant people and their families. One analysis found that more than half of patients seeking abortion spend the equivalent of at least one-third of their monthly income to obtain care.^13^ Studies have also documented that, to pay for abortion care, individuals had to take out loans, sell personal belongings, and forego essential household expenditures such as food, bills, and rent.^14,15,16^

The financial burdens that spending on health services imposes on available household resources—hindering one’s ability to meet basic needs—are often referred to as catastrophic health expenditures (CHEs).^17,18,19^ Although the CHE metric is widely used internationally to evaluate health care financial strain, there is limited literature on catastrophic expenditures for reproductive health care services. Specifically, while it is well established that abortion expenses pose a major barrier to care in the US,^13,20,21^ only 1 study to our knowledge applied the CHE metric to abortion care: Zuniga and colleagues^22^ found that, in most states, the cost of abortion is catastrophic for households earning less than the median monthly income. Nevertheless, their analysis was limited to health care costs and did not account for ancillary expenses, which could be substantial. Building on this work, our primary aim was to estimate the proportion of people presenting for abortion care whose out-of-pocket costs—both direct and indirect—constitute CHEs and to assess whether these costs differ between patients seeking care in their state of residence and those traveling from out of state.

Experiencing a CHE and encountering multiple financial and logistic obstacles when accessing health care might negatively affect one’s psychological well-being. However, the association between access barriers to abortion care and patients’ mental health is understudied.^21,23^ Moreover, research on CHEs often focuses on the financial consequences for individuals but rarely includes adverse mental health as a possible outcome.^24,25^ To address these notable literature gaps and deepen our understanding of the financial and psychological burdens of abortion seeking, our secondary aim was to explore whether incurring abortion-related catastrophic expenditures is associated with adverse mental health symptoms when presenting for care.

## Methods

### Sample

In this cross-sectional study, surveys were administered between January and June 2019 to patients seeking abortion care at 4 clinics in California, Illinois, and New Mexico—all states with Medicaid coverage for abortion for their residents—before the *Dobbs v Jackson Women’s Health Organization* decision ended federal abortion protections. These states and clinics were selected because they served a diverse population, including many patients traveling from other states for care. As detailed elsewhere,^23^ research staff introduced the study to potential participants while they awaited their abortion appointment. Eligible participants were individuals who were pregnant and seeking abortion services, aged 15 to 45 years, proficient in English or Spanish, and not premedicated with narcotics before the abortion. After providing electronic informed consent, participants completed a 20-minute self-administered questionnaire on a tablet device in their preferred language and were compensated for their time with a $30 gift card. The study received approval from the University of California San Francisco Institutional Review Board and followed the Strengthening the Reporting of Observational Studies in Epidemiology (STROBE) reporting guideline for cross-sectional studies.

### Measures

Our primary outcome was abortion-related CHE. We asked participants about their anticipated out-of-pocket direct abortion care cost (“How much do you plan to pay out-of-pocket for your care to end this pregnancy?”) and indirect additional non–health care costs in 6 categories: transportation, accommodation, child care, previous appointments, missed work, and other additional expenses (“Not including the health care costs to end this pregnancy, please tell us about any additional costs associated with coming to the appointment today and then going back home,” eg, “How much are you paying for staying overnight?” and “How much are you paying for child care?”). Participants were instructed to enter 0 if they incurred no costs. We summed both abortion care and additional non–health care costs into 1 variable of total out-of-pocket costs (in 2019 US dollars). To estimate whether these costs were financially catastrophic, we calculated participants’ ability to pay for health spending, defined as the remaining portion of their self-reported monthly household income after subsistence needs such as food or housing had been met.^17,19,26^ According to the US Bureau of Labor Statistics,^27^ the national mean expenditures on food and housing in 2019 accounted for 46% of a household’s income. Therefore, we deducted this amount from participants’ monthly household income to estimate their ability to pay for abortion care. In addition, based on prior research on catastrophic expenditures for reproductive health care^22,28,29^ and the World Health Organization CHE definition,^19,30^ we applied a 40% threshold to create a dichotomous outcome variable: We considered total out-of-pocket costs as CHEs if they exceeded 40% of participants’ estimated ability to pay.

The main exposure was traveling for abortion care, classified as either out of state or in state, based on participants’ state of residence and the clinic location. Additional abortion-seeking variables included the 1-way driving distance to the abortion clinic, in miles, calculated using participants’ residential zip code and the clinic’s zip code; self-reported gestational duration at the time of the abortion appointment; seeking abortion due to fetal medical condition, due to participants’ concerns about their own physical health, and/or because the pregnancy was a result of rape; and planning to use Medicaid to pay for the abortion.

For our secondary aim, we included 3 mental health measures. Stress symptoms were assessed using the Cohen Perceived Stress Scale^31^ (α = 0.62). Anxiety symptoms were measured using the 7-item Generalized Anxiety Disorder scale^32^ (α = 0.94). Depressive symptoms were assessed using the Patient Health Questionnaire–2^33^ (α = 0.86).

Demographic information was collected by self-report. Race and ethnicity were included since racial and ethnic minority individuals are especially encumbered by barriers to abortion care.^3,20^ Categories included Hispanic, Latina, or Latinx; non-Hispanic Asian, Native Hawaiian, or Pacific Islander (hereafter, *Asian, Native Hawaiian, or Pacific Islander*); non-Hispanic Black (hereafter, *Black*); non-Hispanic White (hereafter, *White*); and multiracial or other race and ethnicity (these categories were not broken down further).

### Statistical Analysis

We excluded people who provided no data on expenses and income variables, as we could not calculate their CHEs. To describe the sample and the proportions of people for whom total out-of-pocket costs were estimated as a CHE, we used descriptive statistics. To examine the associations between traveling for abortion care and abortion-related CHEs, we conducted multivariable Poisson regressions and included a fixed effect for recruitment site to account for the clustered structure of the data.^34^ We also conducted multivariable Poisson regressions to examine associations between abortion-related CHEs and adverse mental health symptoms. Covariates for the latter models were selected a priori and followed previous studies on abortion and mental health.^21,35^ We controlled for gestational duration; reason for seeking abortion; relationship with the other person involved in the pregnancy; retrospective pregnancy intentions; history of adverse childhood experiences, depression or anxiety, or problem substance use; and recruitment site. All regression analyses applied multiple imputations, then deletion, using chained equations; all cases were imputed, and those with missing outcome observations were subsequently dropped from the analyses.^36^ Analyses were performed from November 2023 to April 2024, with Stata, version 17 (StataCorp LLC). Two-sided *P* < .05 was considered significant.

## Results

Of the 1092 individuals approached, 846 (78%) agreed to participate; 824 (97%) were eligible and initiated the survey, and of those, 784 (95%) completed it. We excluded 109 people (14%) with missing data on all expenses and income variables, leaving an analytical sample of 675 people. Of the 675 participants, most (374 [55%]) were in their 20s, with a mean (SD) age of 27.33 (6.27) years; 41 (6%) self-identified as Asian, Native Hawaiian, or Pacific Islander; 191 (28%), Black; 162 (24%), Hispanic, Latina, or Latinx; 194 (29%), White; and 84 (12%), multiracial or other race and ethnicity; 3 (1%) did not report their race and ethnicity. The study population closely mirrored the national age and race and ethnicity characteristics of people obtaining a clinic-based abortion before the *Dobbs* decision.^2^ However, individuals who accessed care beyond 12 weeks’ gestation were overrepresented in our sample (196 [29%]) compared with the national rate of 10%.^2^ Additionally, half of the participants (334 [49%]) lived more than 25 miles from the abortion clinic (to convert miles to kilometers, multiply by 1.6), and nearly one-third (212 [31%]) traveled out of state for care (Table 1).

**Table 1.** Sociodemographic, Pregnancy, Abortion-Seeking, and Mental Health Characteristics of People Seeking Abortion in 2019 in California, Illinois, and New Mexico

Characteristic	Participants, No. (%)			*P* value^a^
	In state (n = 463)	Out of state (n = 212)	Total (N = 675)	
**Sociodemographic characteristics**				
Age group, y				
15-19	47 (10)	24 (11)	71 (11)	.06
20-24	120 (26)	49 (23)	169 (25)	
25-29	129 (28)	76 (36)	205 (30)	
30-39	147 (32)	61 (29)	208 (31)	
40-45	20 (4)	2 (<1)	22 (3)	
Self-reported race and ethnicity				
Hispanic, Latina, or Latinx	134 (29)	28 (13)	162 (24)	.04
Non-Hispanic Asian, Native Hawaiian, or Pacific Islander	37 (8)	4 (2)	41 (6)	
Non-Hispanic Black	106 (23)	85 (40)	191 (28)	
Non-Hispanic White	123 (27)	71 (33)	194 (29)	
Multiracial or other^b^	61 (13)	23 (11)	84 (12)	
Missing response	2 (<1)	1 (<1)	3 (<1)	
**Current pregnancy characteristics**				
Current relationship with the other person involved in the pregnancy				
Very or somewhat committed intimate relationship	312 (67)	135 (64)	447 (66)	.97
Friends with benefits, in contact but not in intimate relationship, or other	71 (15)	45 (21)	116 (17)	
Not in contact or no relationship	76 (16)	31 (15)	107 (16)	
Missing response	4 (1)	1 (<1)	5 (1)	
Retrospective pregnancy intentions just before current pregnancy				
Wanted to be pregnant sooner or later	150 (32)	74 (35)	224 (33)	.32
Wanted to be pregnant then	23 (5)	6 (3)	29 (4)	
Never wanted to be pregnant	182 (39)	106 (50)	288 (43)	
Not sure whether pregnancy was wanted	107 (23)	26 (12)	133 (20)	
Missing response	1 (<1)	0	1 (<1)	
**Abortion-seeking characteristics**				
Gestational duration at the time of abortion appointment, wk				
≤12	328 (71)	151 (71)	479 (71)	<.001
13-19	75 (16)	21 (10)	96 (14)	
≥20	60 (13)	40 (19)	100 (15)	
Reasons for seeking abortion^c^^,^^d^				
Fetal medical condition	17 (4)	11 (5)	28 (4)	.01
Pregnancy is result of rape or sexual assault	7 (2)	6 (3)	13 (2)	.02
Concerns about own physical health	77 (17)	35 (17)	112 (17)	.87
Planned payment method for abortion care^d^				
Medicaid, Medi-Cal, or another state-run health insurance program	334 (72)	8 (4)	342 (51)	<.001
Private or employee-sponsored health insurance	34 (7)	4 (2)	38 (6)	
Abortion funds or clinic-based funds	14 (3)	64 (30)	78 (12)	
Out of pocket, own money, or money from other people	110 (24)	188 (89)	298 (44)	
Planned to pay $0 out of pocket for abortion care	317 (68)	17 (8)	334 (49)	<.001
1-Way distance traveled to abortion clinic, mi^e^				
≤25	258 (56)	83 (39)	341 (51)	<.001
26-50	69 (15)	17 (8)	86 (13)	
51-75	35 (8)	3 (1)	38 (6)	
76-100	32 (7)	1 (1)	33 (5)	
>100	69 (15)	108 (51)	177 (26)	
Recruitment site				
Clinic A	69 (15)	137 (65)	206 (31)	NA
Clinic B	180 (39)	3 (1)	183 (27)	
Clinic C	125 (27)	67 (32)	192 (28)	
Clinic D	89 (19)	5 (2)	94 (14)	
**Mental health, childhood adversity, and substance use history^d^**				
History of depression or anxiety diagnosis	140 (30)	49 (23)	189 (28)	.38
Missing response	6 (1)	1 (1)	7 (1)	
History of adverse childhood experiences, any of the following	195 (42)	60 (28)	255 (38)	.15
Lived with someone who had a drinking problem	139 (30)	34 (16)	173 (26)	
Witnessed violence in the neighborhood	123 (27)	44 (21)	167 (25)	
Lived with someone who was mentally ill or depressed	100 (22)	22 (10)	122 (18)	
Lived with someone who served time in jail or prison	93 (20)	27 (13)	120 (18)	
Felt unsupported, unloved, or unprotected at home	89 (19)	28 (13)	117 (17)	
History of problem substance use in past year	177 (38)	78 (37)	255 (38)	.89
Monthly, weekly, or daily use of any illicit or street drugs or prescription drugs for recreational use	71 (15)	25 (12)	96 (14)	
Monthly, weekly, or daily use of ≥4 alcoholic drinks on 1 occasion	152 (33)	70 (33)	222 (33)	

Abbreviation: NA, not applicable.

^a^ Based on Wald tests from logistic regressions adjusted for recruitment site.

^b^ Other race and ethnicity was not broken down further.

^c^ In response to the question, “What are the reasons you are seeking to end this pregnancy?”

^d^ Category data do not add to 100% because participants could check multiple response categories.

^e^ To calculate distance in kilometers, multiply distance in miles by 1.6.

Compared with the 463 patients (69%) who sought care in their state of residence, a significantly higher proportion of the 212 (31%) who traveled from out of state sought abortion at or after 20 weeks’ gestation (40 [19%] vs 60 [13%]; *P* < .001), planned to pay out of pocket for the abortion (188 [89%] vs 110 [24%]; *P* < .001), and lived more than 100 miles away from the clinic (108 [51%] vs 69 [15%]; *P* < .001) (Table 1). Mean (SD) monthly household income was similar for patients who sought care in state vs out of state ($2567 [$2688] vs $2673 [$2442], respectively), as was their ability to pay (mean [SD], $1386 [$1452] vs $1443 [$1319], respectively). Yet, compared with patients who sought care in state, those who traveled from out of state incurred higher total mean (SD) out-of-pocket costs ($1367 [$1929] vs $411 [$606]), including both for abortion care ($838 [$1443] vs $153 [$305]) and for additional non–health care costs ($569 [$974] vs $268 [$447]) (Table 2). The Figure displays the mean out-of-pocket additional non–health care costs for patients overall and those receiving care in state and out of state.

**Table 2.** Household Income, Ability to Pay, Out-of-Pocket Costs, and Abortion-Related CHEs Among People Seeking Abortion in 2019 in California, Illinois, and New Mexico

Variable	Participants		
	In state (n = 463)	Out of state (n = 212)	Total (N = 675)
Monthly household income, mean (SD), $^a^	2567 (2688)	2673 (2442)	2600 (2612)
Monthly household subsistence expenditures of food and housing, mean (SD), $^b^	1181 (1237)	1230 (1124)	1196 (1202)
Ability to pay, mean (SD), $^c^	1386 (1452)	1443 (1319)	1404 (1411)
Out-of-pocket costs, $			
Total^d^			
Mean (SD)	411 (606)	1367 (1929)	711 (1271)
Median (IQR)	189 (10-557)	712 (555-1250)	440 (40-840)
Abortion care^e^			
Mean (SD)	153 (305)	838 (1443)	370 (907)
Median (IQR)	0 (0-87)	540 (405-600)	0 (0-540)
Additional non–health care^f^			
Mean (SD)	268 (447)	569 (974)	363 (674)
Median (IQR)	90 (0-320)	220 (20-700)	120 (0-465)
Total out-of-pocket costs by gestational duration, mean (SD), $			
≤12 wk	327 (494)	873 (1058)	499 (763)
>12 wk	613 (784)	2590 (2857)	1229 (1943)
Participants for whom total out-of-pocket costs were estimated as CHEs, No. (%)^g^	147 (32)	138 (65)	285 (42)
Planning to use Medicaid to pay for the abortion, No./total No. (%)			
Yes	88/334 (26)	5/8 (63)	93/342 (27)
No	59/129 (46)	133/204 (65)	192/333 (58)
Gestational duration, wk			
≤12	83/328 (25)	89/151 (59)	172/479 (36)
>12	64/135 (47)	49/61 (80)	113/196 (58)

Abbreviation: CHE, catastrophic health expenditure.

^a^ There were 6 categorical response categories to the question, “In the last year, what was your total yearly household income before taxes?” (eg, $25 000-$49 000). We selected the midpoint of each category to estimate monthly household income.

^b^ The 2019 national mean subsistence expenditures on food and housing were 46% of household income.^27^

^c^ Calculated by subtracting monthly household subsistence expenditures from monthly household income.

^d^ Included both abortion care and additional non–health care costs.

^e^ In response to the question, “How much do you plan to pay out-of-pocket for your care to end this pregnancy?”

^f^ Included transportation, accommodation, child care, previous appointments, missed work, and other additional expenses. An example question was, “How much are you paying for transportation (tolls, parking, gas, bus fare, airfare, etc)?”

^g^ Catastrophic health expenditures were calculated as total out-of-pocket abortion-related costs greater than or equal to 40% of one’s ability to pay.

**Figure.**
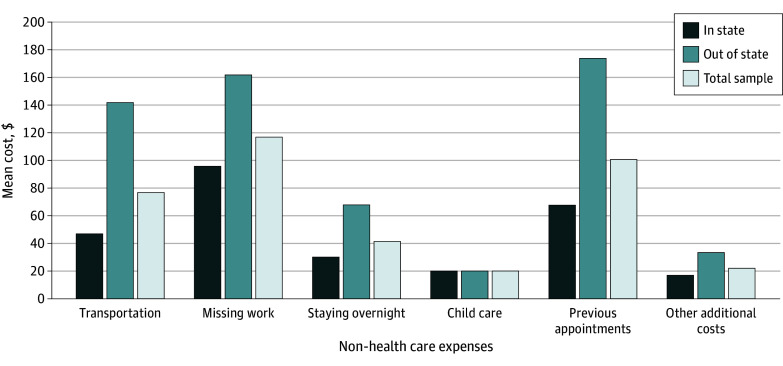
Mean Out-of-Pocket Non–Health Care Costs Among People Seeking Abortion Care In State or Out of State

Total out-of-pocket costs were estimated as CHEs for 285 participants (42%) (Table 2). Patients who traveled out of state for care were significantly more likely to incur abortion-related CHEs (138 of 212 [65%]; adjusted prevalence ratio [APR], 2.24; 95% CI, 1.67-3.00) compared with patients who sought care in state (147 of 463 [32%]). Individuals residing more than 100 miles from the clinic (APR, 2.05; 95% CI, 1.54-2.74) or seeking abortion at or beyond 20 weeks’ gestation (APR, 1.80; 95% CI, 1.30-2.50) were also significantly more likely to incur abortion-related CHEs compared with their counterparts (Table 2 and Table 3).

**Table 3.** Poisson Regression Analyses Assessing Associations Between Abortion-Related CHEs, Out-of-State Travel for Abortion Care, and Other Characteristics Among 675 People Seeking Abortion in 2019 in California, Illinois, and New Mexico

Characteristic	Prevalence ratio (95% CI)			
	Unadjusted	*P* value	Adjusted^a^	*P* value
Traveled out of state for abortion care				
Yes	2.05 (1.62-2.59)	<.001	2.24 (1.67-3.00)	<.001
No	1 [Reference]	NA	1 [Reference]	NA
Abortion-seeking characteristics				
1-Way distance traveled to abortion clinic, mi^b^				
≤25	1 [Reference]	NA	1 [Reference]	NA
26-50	1.05 (0.69-1.59)	.83	1.03 (0.68-1.57)	.88
51-75	0.68 (0.33-1.39)	.29	0.68 (0.33-1.41)	.30
76-100	1.56 (0.92-2.63)	.09	1.29 (0.75-2.23)	.35
>100	2.30 (1.78-2.98)	<.001	2.05 (1.54-2.74)	<.001
Gestational duration at time of abortion appointment, wk				
≤12	1 [Reference]	NA	1 [Reference]	NA
13-19	1.25 (0.89-1.74)	.19	1.28 (0.89-1.85)	.89
≥20	1.95 (1.48-2.57)	<.001	1.80 (1.30-2.50)	<.001
Seeking abortion due to fetal medical condition				
Yes	1.28 (0.76-2.16)	.35	1.11 (0.65-1.90)	.69
No	1 [Reference]	NA	1 [Reference]	NA
Seeking abortion due to concerns about own physical health				
Yes	1.23 (0.92-1.65)	.17	1.22 (0.91-1.65)	.18
No	1 [Reference]	NA	1 [Reference]	NA
Planning to use Medicaid to pay for the abortion				
Yes	1 [Reference]	NA	1 [Reference]	NA
No	2.12 (1.65-2.71)	<.001	2.24 (1.72-2.93)	<.001

Abbreviations: CHE, catastrophic health expenditure; NA, not applicable.

^a^ Derived from Poisson regression models using multiple imputation and adjusted for recruitment site.

^b^ To calculate distance in kilometers, multiply distance in miles by 1.6.

Abortion-related CHE was significantly associated with adverse mental health symptoms when presenting for abortion care: anxiety (APR, 1.13; 95% CI, 1.07-1.19) and depression (APR, 1.25; 95% CI, 1.12-1.39) (Table 4). It was not associated with stress (APR, 1.06; 95% CI, 0.99-1.12). Sensitivity analyses that included all individuals who completed the survey (N = 784) showed substantively similar results throughout.

**Table 4.** Poisson Regression Analyses Assessing Associations Between Abortion-Related CHEs and Adverse Mental Health Symptoms of 675 People Seeking Abortion in 2019 in California, Illinois, and New Mexico

**Symptom**	**Adjusted prevalence ratio (95% CI)^a^**			
	**Partial model^b^**	***P* value**	**Full model^c^**	***P* value**
Perceived stress^d^	1.11 (1.05-1.18)	<.001	1.06 (0.99-1.12)	.03
Anxiety^e^	1.21 (1.15-1.28)	<.001	1.13 (1.07-1.19)	<.001
Depression^f^	1.33 (1.20-1.47)	<.001	1.25 (1.12-1.39)	<.001

Abbreviation: CHE, catastrophic health expenditure.

^a^ Prevalence ratios were derived from Poisson regression models using multiple imputation.

^b^ Adjusted for recruitment site only.

^c^ Adjusted for relationship with the other person involved in the pregnancy, pregnancy intentions, gestational duration, reason for seeking abortion, recruitment site, history of adverse childhood experiences, history of depression or anxiety, and use of illicit or recreational drugs or consumption of 4 or more alcoholic drinks on 1 occasion monthly or more often in the past year.

^d^ Measured with the Cohen Perceived Stress Scale.

^e^ Measured with the 7-item Generalized Anxiety Disorder scale.

^f^ Measured with the 2-item Patient Health Questionnaire.

## Discussion

In our study of individuals presenting for abortion care, 42% were estimated to incur abortion-related CHEs, which were associated with elevated symptoms of stress, anxiety, and depression. These findings shed light on the profound financial and psychological burdens faced by individuals who access abortion care in the US. Because CHE is the result of high out-of-pocket costs combined with low ability to pay, patients accessing abortion care are at particularly high risk for CHE, with studies showing that most live on a low income^37^ and many lack health insurance.^1,2,3,4^ Extensive research also demonstrates that the Hyde Amendment contributes to major financial hardships, disproportionately affecting people with low incomes, racial and ethnic minority individuals, and other marginalized populations.^13,15,20,38^ Facing CHE can be devastating and long-lasting for these households, triggering high levels of debt, financial insecurity, worsened health outcomes, and increased impoverishment.^39,40,41^ Our finding that abortion-related CHEs were associated with adverse mental health symptoms when presenting for abortion care further underlines not only the financial but also the potential psychological consequences of abortion restrictions.

Furthermore, we found that 65% of participants who traveled out of state for care were estimated to incur abortion-related CHEs. Patients from out of state incurred higher costs, as expected since such patients are ineligible for state Medicaid coverage and they face longer care-seeking processes.^1^ Thus, those who traveled from out of state for care in this study were significantly more likely to incur CHEs, despite having a similar ability to pay as patients who sought care in state. Moreover, the proportion of patients traveling from out of state who were estimated to incur abortion-related CHEs was high regardless of gestational duration or whether they planned to pay with Medicaid. These results are consistent with numerous studies that have documented the logistical and financial challenges of out-of-state travel for abortion care.^12,42,43,44^ Yet, to our knowledge, our study is the first to apply the CHE metric in that regard, emphasizing the added burden that out-of-state travel places on patients seeking abortion care.

Overall, our study underscores the financial and psychological strain faced by patients who access abortion care when paying out of pocket. It supports the critical role of Medicaid in reducing these costs,^3,45^ as most participants in our study who sought care in state planned to use Medicaid and anticipated no out-of-pocket expenses for their abortion care. However, 32% of patients seeking care in state were still estimated to incur CHEs. This aligns with prior research, which indicates that some Medicaid-eligible individuals may encounter barriers to accessing coverage due to complex reimbursement processes and confidentiality concerns.^13,46^ Given that health insurance coverage can mitigate the risk of CHE,^25,47,48,49^ repealing the Hyde Amendment and any other state-level insurance restrictions on abortion is crucial to ensuring equitable access to care.

Yet, health insurance addresses only the costs of the abortion itself and does not usually cover, or is not used for, ancillary non–health care costs such as accommodation and transportation. Our results indicate that both direct and indirect expenses are substantial. Moreover, since our study was conducted, the number of patients seeking abortion care who incur catastrophic expenditures is likely even higher due to a growing proportion of people traveling out of state for care since the *Dobbs* decision.^50,51,52,53^ This increase is found particularly in states bordering those with abortion bans, such as Illinois and New Mexico^53^—2 states where our data were collected. Therefore, alongside promoting and expanding insurance coverage, policy efforts should prioritize ensuring accessible and timely abortion care to alleviate the financial burden faced by individuals and their households, regardless of their state of residence.

### Limitations

This study has several limitations. First, our recruitment clinics were all located in states with Medicaid coverage for abortion, which is accessible only to state residents. Two recruitment sites also had very few participants who traveled from out of state for care, limiting the diversity of our sample and potentially affecting the generalizability of our results. Future research should explore the financial burden experienced by people trying to access abortion care from a broader range of clinics and in states without Medicaid coverage for abortion.

Second, our calculation of CHE relied on self-reported data collected at the time of presenting for care, before all expenses were incurred. While our approach offers valuable insights, measures of both income and out-of-pocket costs may be biased and do not account for unanticipated financial assistance, expenses, or savings that could occur during or after the visit. Many participants also had missing income data, which is difficult to report accurately, particularly for young people and those with inconsistent incomes. Although we used multiple imputation methods to address missing data, these methods cannot fully eliminate bias introduced by data that are not missing at random.

Third, our cross-sectional design prevents us from establishing causality between abortion-related CHE and adverse mental health symptoms. While we controlled for mental health history, we cannot rule out the possibility that people with more adverse mental health symptoms are more likely to incur CHEs. Additionally, we did not assess long-term financial and psychological repercussions or coping mechanisms over time. Future research should incorporate longitudinal designs to provide a more comprehensive understanding of the financial and psychological outcomes of abortion care.

Fourth, the context of abortion care has changed substantially since fielding this study. The proliferation of telehealth access to medication abortion has lowered the cost of abortion and, for some individuals, reduced or even eliminated travel-related costs.^6,54^ Future research should explore these significant developments.

## Conclusions

This pre-*Dobbs* cross-sectional study found that 42% of patients seeking abortion care were estimated to incur abortion-related catastrophic expenditures, which were associated with adverse mental health symptoms when patients presented for care. Compounded by higher direct and indirect costs, individuals who needed to travel from out of state to reach care were significantly more likely to incur CHEs. These financial and psychological burdens encountered by patients who seek abortion care are likely even worse in the current post-*Dobbs* context, when more people must travel longer distances and out of their state of residence to access care.^50,51,52,53^ The findings suggest a need to expand insurance coverage to ensure equitable access to abortion care, irrespective of people’s state of residence.

## Notes

Supplement.
Data Sharing Statement
