# Supplementary material for: Catastrophic Health Expenditures for In-State and Out-of-State Abortion Care
Source: JAMA Netw Open. 2024 Nov 8;7(11):e2444146. doi: 10.1001/jamanetworkopen.2024.44146 (PMC11549660; doi:10.1001/jamanetworkopen.2024.44146)
Supplement: Supplement. — Data Sharing Statement [file jamanetwopen-e2444146-s001.pdf]

Data Sharing Statement

Wasser. Catastrophic Health Expenditures for In-State and Out-of-State Abortion Care. *JAMA Netw Open*. Published November 08, 2024. doi:10.1001/jamanetworkopen.2024.44146

Data
Data available: No

Additional Information
Explanation for why data not available: Data will not be made available due to patient privacy concerns given the stigmatized nature of abortion care.
